# Not Your Typical Simulation Workshop: Using LEGOs to Train Medical Students on the Practice of Effective Communication

**DOI:** 10.7759/cureus.2094

**Published:** 2018-01-21

**Authors:** Dimitrios Papanagnou, Hyunjoo Lee, Carlos Rodriguez, Xiao Chi C Zhang, Joshua Rudner

**Affiliations:** 1 Department of Emergency Medicine, Thomas Jefferson University

**Keywords:** medical education, teamwork, simulation, communication, medical student

## Abstract

As students in the health professions transition from the classroom into the clinical environment, they will be expected to effectively communicate with their team members and their patients. Effective communication skills are essential to their ability to effectively contribute to their clinical team and the patient care they deliver. The authors propose an interactive workshop that can support students’ deliberate practice of communication skills.

The authors designed a simulation workshop that affords students the opportunity to practice their communication and peer-to-peer coaching skills. Using LEGOs, a one-hour workshop was conducted with medical students. Students were divided into groups of two. Each student took on a different role: teacher or builder. Teachers were tasked with instructing builders on how to construct a pre-made LEGO structure, not allowing builders to look at the structure. A group debriefing followed to evaluate the activity and explore the themes that emerged.

Twenty first-year medical students and 25 fourth-year medical students participated in this activity. Most groups were successful in reproducing the pre-made structure. Groups that pre-briefed before building were most successful. Unsuccessful groups did not define orientation or direction in mutually understood terms, resulting in the creation of an incorrect mirror image of the structure – a common phenomenon seen during the teaching of procedures in the clinical learning environment.

The workshop was well received. Students made requests to have similar sessions throughout their training to better support the development of effective communication skills. The workshop can easily be applied to other specialties to assist with procedural skills instruction or in workshops focusing on effective communication.

## Introduction

Medical students generally have a rather drastic transition from being pre-clinical to becoming clinical students. Part of the transition to a clinical student involves interacting with patients in the clinical environment. Effective communication skills are paramount in thriving as a contributing member of the team. As students progress into residency, they are expected to take on the teaching role. Studies estimate that residents spend up to 20% of their time in teaching [1]. Pedagogical interventions focusing on communication have been shown to enhance confidence with teaching, increase self-reported use of teaching behaviors, and improve evaluations of residents’ teaching effectiveness [2,3].

Simulation offers medical students the chance to learn about how they learn and also to develop their own teaching skills [4]. A study of pediatric fellows using a simulation-based session to provide teacher-training showed improvements in confidence and self-rated teaching abilities [5]. While well described in supporting the practice of technical procedures, simulation can also improve providers’ teamwork and communication skills [6-8]. A study used a LEGO® (The LEGO group, Denmark) simulation to improve communication skills between first-year medical students and simulated patients [9]. In the current study, we expanded upon the idea of using LEGO simulations for medical teaching. We developed a one-hour workshop for medical students to practice their teaching of procedural skills via a LEGO-building simulation to foster the deliberate practice of effective communication skills.

## Technical report

Setting and participants

The workshop was implemented with two groups of medical students: a random sample of first-year medical students during their medical school orientation week and a random sample of fourth-year medical students during their fourth year.

Student selection

Students were randomly selected from two convenience samples. A group of first-year medical students was compared to a group of fourth-year medical students to evaluate for any possible differences in the LEGO activity; this decision was made to deliberately compare medical students at the start of their medical training to medical students nearing the end of their undergraduate medical education.

Materials required

Each student pair received one sandwich bag of a pre-made LEGO structure, and another sandwich bag of the necessary pieces needed to recreate the pre-made structure.

Group structure

Each group of students (i.e., first- and fourth-year groups) was divided into dyads, allowing each member to take on a different role: teacher or builder. The task of the teacher was to instruct his/her peer how to assemble a pre-made LEGO structure with all the pieces needed, without ever viewing the structure. The task of the builder was to successfully assemble a carbon copy of the LEGO structure relying only on the verbal information provided by his/her student peer. Workshop facilitators observed the building process and made observations that would assist in the debriefing of the activity. The activity was then repeated, during which students had the opportunity to change roles.

Detailed activity description

**Figure 1:**
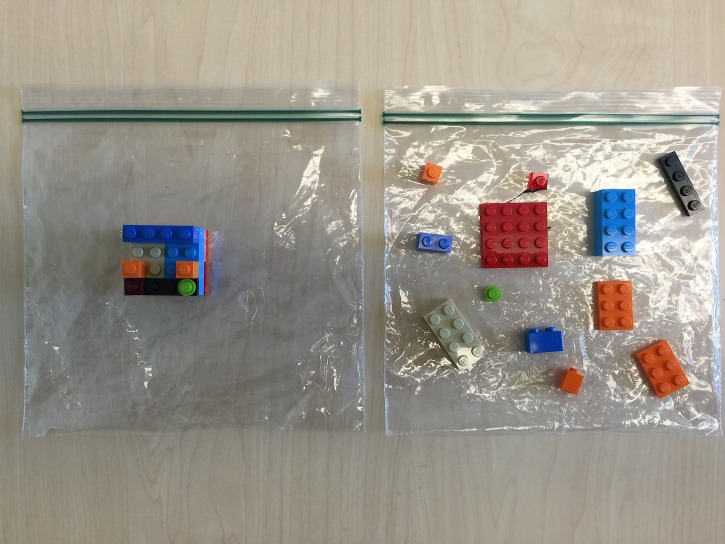
Pre-made bags for distribution to each group. One bag contained the pre-made LEGO structure; the other bag contained the unassembled LEGO pieces.

Prior to the workshop, the number of dyads was anticipated to secure the appropriate amount of workshop materials. For each dyad, 30 LEGO pieces were set aside. It was essential that there were two of each piece; this ensured that there were two identical sets of LEGO sets per group. With one set of LEGO pieces, a random structure was created using 15 pieces, and placed in one of the plastic bags. The 15 remaining LEGO pieces, of the other identical set, were loosely placed in another plastic bag, and used to create a replica of the original structure (Figure 1). As many pairs of these bags as necessary should be created to run the workshop. Structures and LEGO pieces can and should vary from group to group.

Each group was provided with a set of two bags: one bag of the pre-made structure and another bag of the pieces required to create that structure. The pre-made LEGO structure was given to the assigned ‘teacher.’ The loose LEGO pieces, needed to make the pre-made structure, were given to the assigned ‘builder.’ Builders were prohibited from looking at the pre-made structure, and could only rely on verbal information during the assembly.

Teachers and builders sat with their backs against one another. Teachers were encouraged to use as much descriptive information as possible without ever showing the builders the structure at hand. Workshop facilitators walked around the learning space, navigating the dyads’ progress, and recorded observations. Attention was given to challenges encountered and how they were effectively or ineffectively navigated. Time permitted, which allows the activity to be repeated, during which student roles were flipped.

Workshop evaluation

The authors used Stephen Brookfield’s Critical Incident Questionnaire (CIQ) to obtain written feedback about the LEGO workshop. Brookfield’s CIQ was originally designed to elicit anonymous, concrete feedback from students about their experience with classroom educational activities [10]. The questionnaire included the following five questions:

a)     “At what moment did you feel most engaged?”

b)    “At what moment did you feel most distanced?”

c)     “What action did you find most affirming and helpful?”

d)    “What action did you find most puzzling or confusing?”

e)     “What surprised you the most?”

Comments captured from the CIQ for both first-year and fourth-year medical students were evaluated through open-axial qualitative analysis performed by the two of the authors (D Papanagnou, H Lee), who have extensive experience with qualitative research.

Results

**Figure 2:**
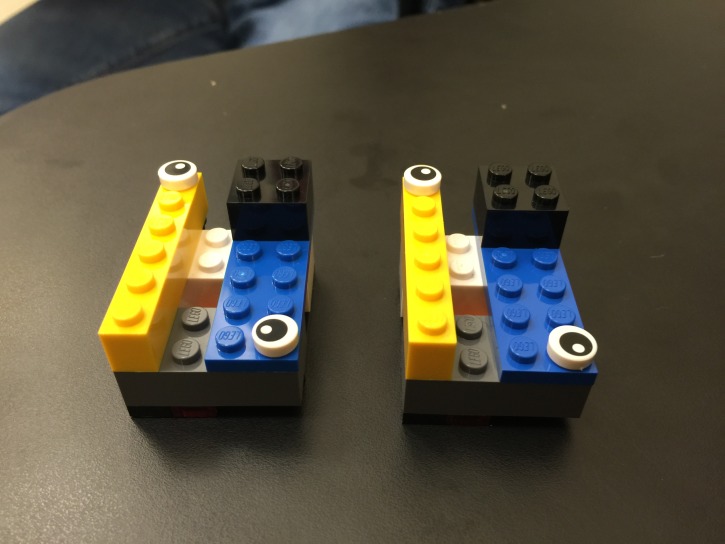
Successful completion of the activity: two identical LEGO structures.

Twenty first-year medical students and 25 fourth-year medical students participated in this activity. Most groups were successful in reproducing the pre-made structure (Figure 2).

**Figure 3:**
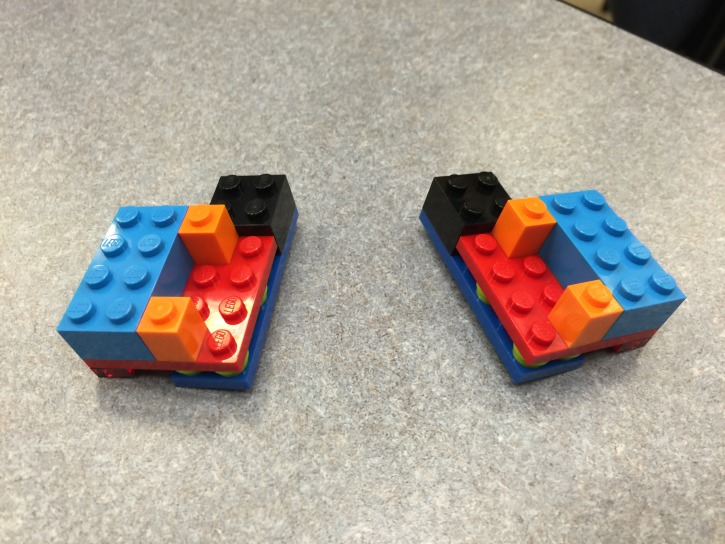
Example of an incorrect assembly of an enantiomer structure, a mirror image of the original structure.

A finding was noted in several groups that were initially thought to have completed the task correctly. In these groups, the structures were not identical to one another; rather, they were mirror images of one another (Figure 3). Three groups in the first-year class created these enantiomers, whereas no group in the fourth-year class made enantiomers. During the debriefing, it was discovered that these first-year student groups did not define orientation and direction in terms that were mutually understood by the ‘teacher’ and the ‘builder.’ Several parallels were made with the spatial understanding that is intrinsic to procedures in the clinical environment, including ultrasound and ultrasound-guided applications.

Student feedback from the CIQ yielded several themes (Table 1). Overall, the first- and fourth-year medical students commented on the challenges of effective communication and the importance of a shared mental model and language. Both groups felt most engaged during the actual participation of the activity, particularly when they were receiving confirmatory cues that the structure was being built correctly. Most students in both groups did not feel distanced during the activity. One first-year student, however, did comment that he/she felt distanced when the facilitators used specific anatomical examples to correlate the importance of spatial orientation, as first-year students had not had any formalized training in anatomy. The majority of the students found the debriefing most helpful, particularly when they were able to discuss different strategies, including effective communication (i.e., closed-loop communication). Fourth-year students commented on how their use of closed-loop communication was the most affirming (or helpful) part of the activity.

**Table 1:** Thematic codes generated from responses to the Brookfield Critical Incident Questionnaire.

Question	First-Year	Fourth-Year
Most Engaged	Participatory involvement Relevance to clinical practice Effective communication Feedback on performance	Pre-briefing and shared language Strategies to effective communication Engaging in healthy competition
Most Distanced	First exposure in communication Visual-spatial orientation Correlations to clinical anatomy Challenges to effective communication *Most students did not feel distanced	Sub-optimal communication strategies Difficulty establishing a shared language Intrinsic challenge of the activity *Most students did not feel distanced
Most Affirming/Helpful	Correlations to real clinical applications Debriefing of activity Discussing communication strategies Shared mental models	Relevance to clinical practice Establishing shared language Effective communication strategies
Most Puzzling/Confusing	Lack of shared mental model Challenges to effective communication *Most students were not puzzled or confused	Difficulty establishing shared language Limited availability of (visual) information *Most students were not puzzled or confused
Most Surprising	Challenge of effective communication Clinical relevance of activity Difficulty in teaching effectively Engagement of activity	Challenge of effective communication Importance of effective communication Importance of shared language Need for multi-modal informational cues Challenge of the activity

Both groups felt most confused when trying to create a language to direct one another throughout the activity. First-year students were most surprised to find out how relevant the activity was when the communication skills employed were correlated with clinical activities (i.e., communicating with patients). Fourth-year students were most surprised by how difficult the activity actually was, but how effective closed-loop communication assisted in completing the activity correctly.

## Discussion

Our workshop encourages participants to appreciate the importance of effective and specific communication strategies that can potentially lend to effective teaching, procedural instruction, and communication with team members and patients. Because LEGO ‘teachers’ in the workshop are only allowed to provide verbal cues, and ‘builders’ are only allowed to use auditory information to construct their structures, participants have the unique opportunity to experiment with and reflect on their communication styles (i.e., phrases, approaches, word choices) that can lead to more successful outcomes. Additionally, ‘builders’ have the opportunity to empathize with their peers who are struggling to build their LEGO structures with minimal information (i.e., visual information, spatial orientation). By its intrinsic design, the activity prompts learners to speak-up and ask for help when they are challenged.

By repeating the activity and having the student rotate through the tasks of being both a teacher and a builder, the student is provided with: a) the ability to experience procedural instruction from multiple perspectives; b) the chance to be mindful of one’s observations as he/she teaches and guides with descriptive information; and c) the time to empathize with the challenges ascribed to both roles.

The educational philosophy of this workshop is rooted in deliberate practice: a critical process for the development of mastery expertise. Ericsson, et al. proposed that the number of hours spent in deliberate practice is an important determinant of one’s level of expertise [11]. This workshop creates the space for students to safely practice procedural instruction to their peers and receive formative feedback from an observer on their performance.

A principle-based approach to teaching technical skills has been shown to be more effective than the traditional “see one, do one, teach one” approach. McLeod, et al. developed a checklist of principles for teaching procedural and technical skills [12]. These include planning ahead, observing the learner in action, allowing for practice, and providing feedback and an opportunity for self-assessment. The simulated LEGO workshop embraced this principle-based approach. Likewise, students commented that they felt the most engaged while actively participating in the building process, and found the creation of a shared language and the utilization of closed-loop communication to be most helpful.

Debriefing and reflection is also a valuable component of the experiential learning process, allowing all learners, teachers and builders alike, to reflect on their experience and develop and integrate these insights into later behavior in the clinical learning environment [13,14]. Based on comments shared during the group debriefing, groups that planned ahead and pre-briefed were most successful. Examples of this included having the builder itemize all the pieces in his/her bag; identifying all of the salient pieces in the structure; defining orientation and direction (i.e., what is right versus left) before the building process began; and creating an atmosphere that was conducive to pausing, speaking up, and asking for repeated instruction.

Even in groups where specific and descriptive information was successfully provided to the builder, several students commented on the challenge of not being able to integrate visual information and visual cues into the building process. This highlights that several students will require a multimodal approach to procedural instruction. The concerns regarding the challenge of effective communication were voiced by both first- and fourth-year medical students; fourth-year students, however, performed better on the LEGO activity than their first-year counterparts. This may suggest the importance of integrating opportunities to discuss, practice, and reinforce effective communication strategies across the entire curriculum, and as early as the first year.

Limitations

While our workshop was originally designed to be an innovative workshop, findings are limited by the low sample size of students who underwent this activity. The first- and fourth-year students chosen represent convenience samples. First-year students chose to this attend this workshop from a list of educational offerings during their medical school orientation week in the Fall. Fourth-year students who participated in the workshop were randomly selected from students participating in the required senior Emergency Medicine clerkship during orientation day in the Fall. In addition, quantitative estimates of how long it took each dyad to successfully complete the LEGO activity was not recorded; subsequent iterations of this workshop can potentially examine the time it takes teams to successfully complete the task, and correlating temporal estimates with year of training and thematic review of communication patterns between team members.

## Conclusions

The communication workshop was well received by our medical students. Using LEGOs, the workshop provided students with the opportunity to practice verbal cueing, procedural coaching techniques, and effective debriefing in a non-clinical, non-threatening, fun, and practical manner. Students made requests to have additional, similar sessions across the academic year. Our workshop can easily be replicated in any specialty to assist in teaching the concepts of procedural skills instruction and effective communication skills, such as closed-loop communication and situational awareness.
